# Recurrent Foreign Body Ingestion in a Psychiatric Patient: A Conservative Approach

**DOI:** 10.7759/cureus.89812

**Published:** 2025-08-11

**Authors:** Clara L Voltarelli, Beatriz Kneif Reis, Lucas dos Santos Siviero, Caroline de Lima Bertuol, Larissa Hermann de Souza Nunes

**Affiliations:** 1 Internal Medicine, Hospital Universitário Cajuru, Curitiba, BRA; 2 Inernal Medicine, Hospital Universitario Cajuru, Curitiba, BRA; 3 Internal Medicine, Pontifícia Universidade Católica do Paraná (PUC-PR), Curitiba, BRA; 4 Internal Medicine, Faculdades Pequeno Príncipe, Curitiba, BRA; 5 Internal Medicine, Hospital Universitario Cajuru, Curitiba, BRA

**Keywords:** conservative vs surgical management, deliberate foreign body ingestion, eating disorder, psychiatric comorbidity, self-injurious behavior

## Abstract

Foreign body ingestion in psychiatric patients is a challenging condition, often linked to severe complications and significant morbidity. We report the case of a 33-year-old woman with bipolar affective disorder and borderline personality disorder who presented with abdominal pain, melena, vomiting, and anorexia. Imaging and endoscopy revealed multiple ingested nails. The patient had a history of four previous surgical interventions for similar episodes, resulting in extensive abdominal adhesions. After a failed endoscopic removal, conservative management was chosen, with close clinical and radiological monitoring. She was discharged after 18 days without retained foreign bodies or complications. This case highlights the importance of individualized management in psychiatric patients with recurrent foreign body ingestion. Although surgery is sometimes necessary, its risks increase with repeated laparotomies. Conservative treatment can be a safe alternative, especially when endoscopy fails. A multidisciplinary approach is essential to evaluate risks and guide the choice between invasive and non-invasive strategies for optimal outcomes.

## Introduction

Deliberate foreign body ingestion represents a significant clinical challenge, particularly among patients with psychiatric disorders, where such behavior is often associated with impulsivity, self-harm, or suicidal ideation. It is estimated that up to 80% of cases occur in individuals with a psychiatric history, especially those diagnosed with borderline personality disorder, bipolar affective disorder, schizophrenia, and mild intellectual disability [1,2].

Sharp and metallic objects, such as nails, blades, and screws, are among the most commonly ingested items in intentional cases and carry a high risk of complications, including gastrointestinal perforation, bleeding, obstruction, and peritonitis [3]. While most accidentally ingested foreign bodies pass spontaneously, deliberate and recurrent ingestion-particularly of sharp or voluminous objects-often requires endoscopic or surgical intervention [4].

Management becomes more complex in patients with multiple prior abdominal surgeries, such as laparotomies, due to recurrent foreign body ingestion, as these cases often involve a *hostile abdomen* characterized by extensive adhesions and increased risk of intra- and postoperative complications [5]. In such scenarios, conservative management may be a viable alternative when the patient is clinically stable, with no signs of perforation, sepsis, or intestinal obstruction [6].

Diagnostic assessment should include imaging studies such as plain radiographs and computed tomography scans, which help locate the foreign bodies, monitor their progression, and detect early signs of complications. Upper gastrointestinal endoscopy also plays a key role in both diagnosis and treatment, and is indicated when endoscopic removal is feasible and safe [7].

From a psychiatric perspective, voluntary ingestion of objects may reflect chronic or impulsive self-harming behavior, requiring a multidisciplinary therapeutic plan that integrates clinical, surgical, and ongoing psychiatric support. Follow-up in specialized services, such as Psychosocial Care Centers (CAPS), is essential to ensure treatment adherence and prevent recurrence [2].

Recurrence is common in these cases, reinforcing the need for risk-reduction strategies and structured psychotherapeutic approaches such as dialectical behavior therapy (DBT), combined with the appropriate use of mood stabilizers and antipsychotics tailored to the individual case [8].

## Case presentation

A 33-year-old woman with a diagnosis of bipolar affective disorder and borderline personality disorder was under regular follow-up at a CAPS facility. She had a history of four previous suicide attempts by foreign body ingestion, each resulting in gastrointestinal complications that required surgical intervention via laparotomy.

Her ongoing pharmacological regimen included biperiden 2 mg/day, haloperidol (two intramuscular injections every 15 days), valproic acid 500 mg (1-0-2), lithium 300 mg (1-0-2), and diazepam. She reported good adherence to treatment.

The patient presented to the emergency department reporting ingestion of a large quantity of nails approximately 30 days prior. In the five days leading up to admission, she experienced progressively worsening abdominal pain, altered bowel habits, and episodes of melena. On examination, she appeared in good general condition, with diffuse abdominal tenderness and mild discomfort, but without signs of peritonitis.

**Figure 1:**
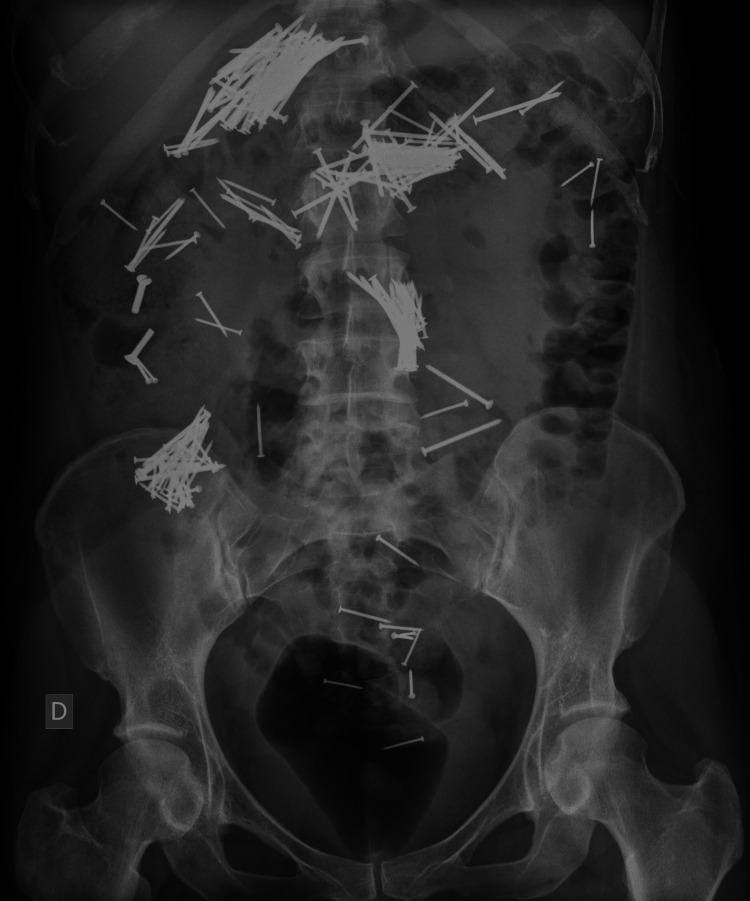
Abdominal X-ray upon arrival. Supine abdominal radiograph obtained upon presentation shows numerous metallic foreign bodies (nails) dispersed throughout the colon and jejunal loops, indicating widespread gastrointestinal distribution.

**Figure 2:**
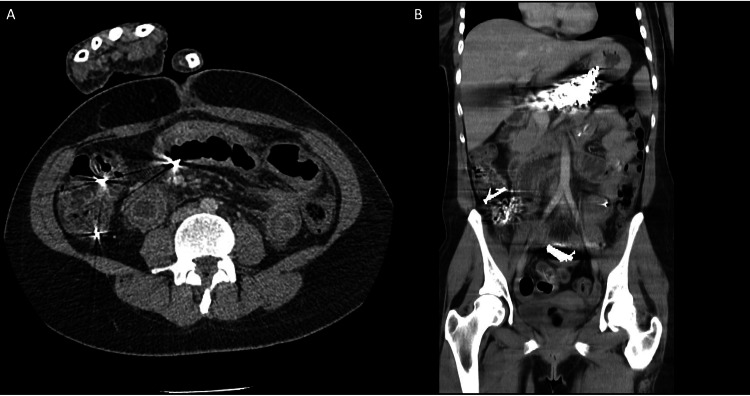
Abdominal CT scan demonstrating extensive ingestion of metallic foreign bodies. (A) Axial view showing numerous metallic objects within the gastric lumen and small bowel loops, with evident segmental wall thickening of the small intestine and adjacent fat stranding. (B) Coronal reconstruction revealing widespread distribution of foreign bodies throughout the stomach, small intestine, and colon. Segmental bowel wall thickening and surrounding fat stranding are noted, particularly in portions of the jejunum and colon, suggestive of an inflammatory response secondary to mucosal trauma. No pneumoperitoneum is identified.

**Figure 3:**
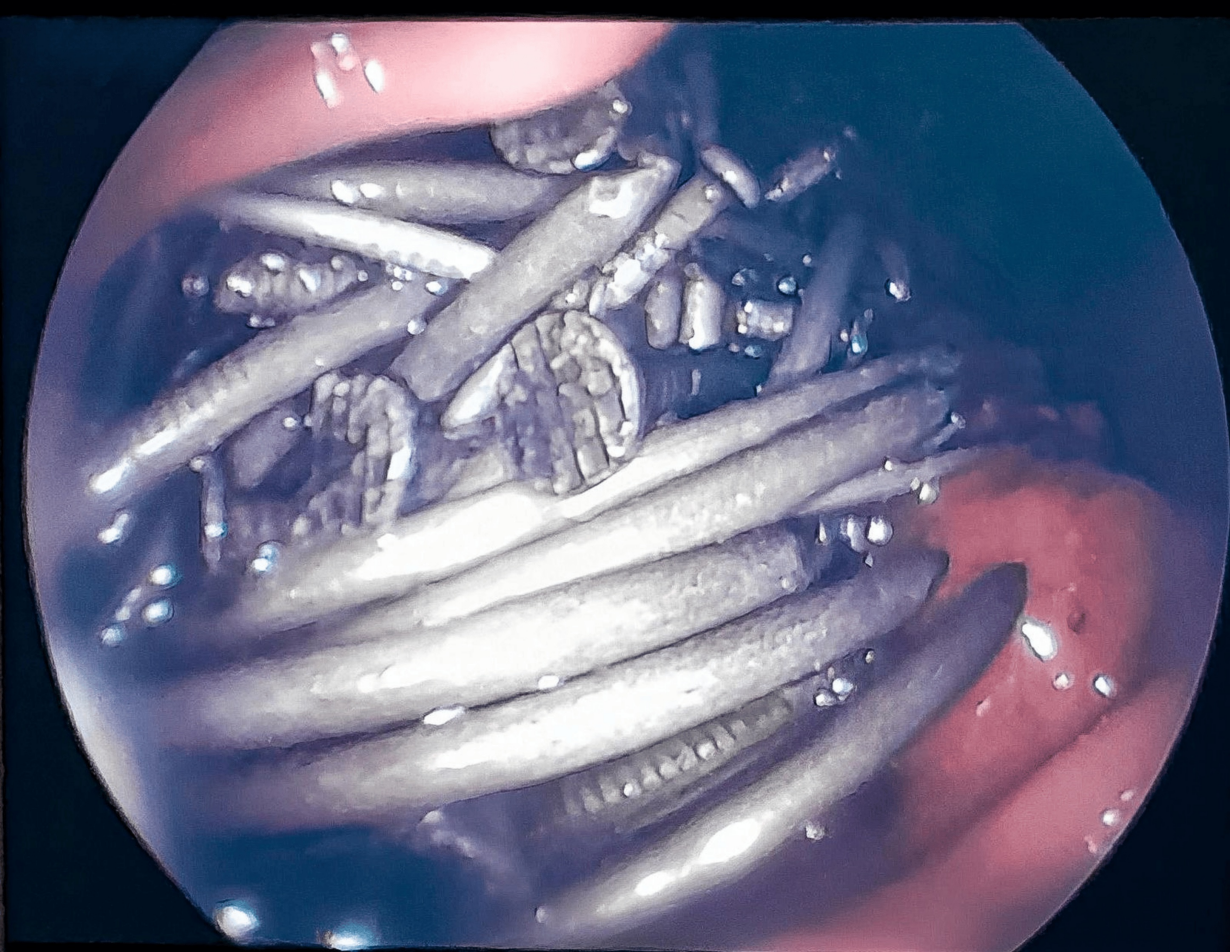
Upper gastrointestinal endoscopy showing multiple metallic foreign bodies in the stomach. Endoscopic view revealing a large accumulation of ingested nails occupying the gastric lumen. The quantity and arrangement of the objects rendered endoscopic removal unfeasible due to the high risk of mucosal injury.

Upon arrival, a supine abdominal X-ray demonstrated multiple radiopaque foreign bodies, consistent with ingested nails, dispersed throughout the colon and jejunal loops, indicating widespread gastrointestinal distribution (Figure 1). Subsequently, a CT scan was performed, revealing a large volume of metallic foreign bodies within the stomach, colon, and small bowel loops (Figure 2). Segmental bowel wall thickening and adjacent fat stranding were also noted, suggestive of an inflammatory response likely secondary to mucosal trauma from the ingested material. No signs of pneumoperitoneum were identified, indicating the absence of gastrointestinal perforation at that time. Following imaging, upper gastrointestinal endoscopy confirmed a large number of nails in the stomach, rendering endoscopic removal unfeasible (Figure 3).

Given the patient's initial clinical stability and her history of multiple laparotomies with significant intra-abdominal adhesions (i.e., a hostile abdomen), a conservative management strategy was adopted. This included analgesia and close clinical monitoring. The decision to delay surgical intervention was supported by the absence of signs indicating acute complications.

Throughout hospitalization, the patient remained hemodynamically stable, with gradual symptomatic improvement and spontaneous, progressive passage of the ingested nails. Serial abdominal radiographs confirmed the distal progression of the foreign bodies, with no evidence of obstruction or perforation.

After 18 days of inpatient care, complete elimination of the foreign bodies was documented without complications. The patient was discharged with reinforced recommendations for intensive psychiatric follow-up at her CAPS unit, with a focus on treatment adherence and prevention of future self-harming behavior.

## Discussion

Voluntary foreign body ingestion represents a complex clinical scenario with significant implications for both medical and psychiatric management, particularly in patients with severe mental disorders. Most cases are associated with underlying psychiatric conditions, especially borderline personality disorder, bipolar affective disorder, schizophrenia, and mild intellectual disability [1,2]. In the case described, the patient presented with significant psychiatric comorbidities, namely bipolar disorder and borderline personality disorder, both of which are strongly associated with impulsivity and recurrent self-injurious behavior.

The ingestion of sharp metallic objects such as nails carries a high risk of serious gastrointestinal complications, including perforation, bleeding, obstruction, and peritonitis [3]. While most accidentally ingested foreign bodies are expelled spontaneously, deliberate and repeated ingestion, particularly of sharp or voluminous objects, often requires intervention [4]. Nonetheless, clinical management should be individualized, taking into account the patient’s hemodynamic status, the type and quantity of objects ingested, and their surgical history.

In this case, the patient had undergone four prior laparotomies due to similar episodes, leading to a *hostile abdomen* with dense adhesions and increased surgical risk [5]. Given this context, and in the absence of signs of perforation, sepsis, or bowel obstruction, a conservative approach was adopted. This decision aligns with current literature, which supports non-operative management in hemodynamically stable patients without complications [6].

Serial abdominal radiographs played a crucial role in monitoring the progression of the foreign bodies through the gastrointestinal tract. Initial CT imaging and upper endoscopy were essential for anatomical assessment and exclusion of acute complications. However, endoscopic removal was not feasible in this case due to the volume of ingested material [7].

From a psychiatric standpoint, recurrent self-injurious behavior, including foreign body ingestion, is expected in patients with certain personality disorders. Continued intensive psychiatric follow-up through community-based services such as CAPS, along with pharmacological management using mood stabilizers, antipsychotics, and anxiolytics, is key to reducing recurrence [2]. Furthermore, structured psychotherapeutic approaches, particularly DBT, have shown strong evidence in managing patients with similar clinical profiles [8].

## Conclusions

In summary, this case highlights the complexity of managing recurrent foreign body ingestion in psychiatric patients. It demonstrates that conservative treatment, when carefully selected, can be a safe and effective alternative, even in cases of massive ingestion. A multidisciplinary approach encompassing clinical, surgical, and psychiatric teams is essential to provide comprehensive, patient-centered care that minimizes risks and supports long-term recovery.

This case also underscores the importance of individualized risk-benefit assessment, particularly in patients with a history of multiple abdominal surgeries and complex psychiatric comorbidities. Prompt imaging and vigilant clinical monitoring are critical to guide therapeutic decisions and to avoid unnecessary surgical interventions.

Finally, sustained psychiatric engagement, including structured psychotherapy and adherence to pharmacological treatment, remains the cornerstone of preventing recurrence and improving long-term prognosis in this vulnerable population.
